# Cationic Polyethyleneimine (PEI)–Gold Nanocomposites Modulate Macrophage Activation and Reprogram Mouse Breast Triple-Negative MET-1 Tumor Immunological Microenvironment

**DOI:** 10.3390/pharmaceutics14102234

**Published:** 2022-10-19

**Authors:** Vladimir Mulens-Arias, Alba Nicolás-Boluda, Florent Carn, Florence Gazeau

**Affiliations:** 1 Matière et Systèmes Complexes (MSC), Université Paris Cité, CNRS, 45 rue des Saints Pères, 75006 Paris, France; 2 Integrative Biomedical Materials and Nanomedicine Lab, Department of Medicine and Life Sciences (MELIS), Pompeu Fabra University, PRBB, Carrer Doctor Aiguader 88, 08003 Barcelona, Spain

**Keywords:** cationic nanostructure, gold, nanoparticles, tumor microenvironment, immune cells, polyethyleneimine

## Abstract

Nanomedicines based on inorganic nanoparticles have grown in the last decades due to the nanosystems’ versatility in the coating, tuneability, and physical and chemical properties. Nonetheless, concerns have been raised regarding the immunotropic profile of nanoparticles and how metallic nanoparticles affect the immune system. Cationic polymer nanoparticles are widely used for cell transfection and proved to exert an adjuvant immunomodulatory effect that improves the efficiency of conventional vaccines against infection or cancer. Likewise, gold nanoparticles (AuNPs) also exhibit diverse effects on immune response depending on size or coatings. Photothermal or photodynamic therapy, radiosensitization, and drug or gene delivery systems take advantage of the unique properties of AuNPs to deeply modify the tumoral ecosystem. However, the collective effects that AuNPs combined with cationic polymers might exert on their own in the tumor immunological microenvironment remain elusive. The purpose of this study was to analyze the triple-negative breast tumor immunological microenvironment upon intratumoral injection of polyethyleneimine (PEI)–AuNP nanocomposites (named AuPEI) and elucidate how it might affect future immunotherapeutic approaches based on this nanosystem. AuPEI nanocomposites were synthesized through a one-pot synthesis method with PEI as both a reducing and capping agent, resulting in fractal assemblies of about 10 nm AuNPs. AuPEI induced an inflammatory profile in vitro in the mouse macrophage-like cells RAW264.7 as determined by the secretion of TNF-α and CCL5 while the immunosuppressor IL-10 was not increased. However, in vivo in the mouse breast MET-1 tumor model, AuPEI nanocomposites shifted the immunological tumor microenvironment toward an M2 phenotype with an immunosuppressive profile as determined by the infiltration of PD-1-positive lymphocytes. This dichotomy in AuPEI nanocomposites in vitro and in vivo might be attributed to the highly complex tumor microenvironment and highlights the importance of testing the immunogenicity of nanomaterials in vitro and more importantly in vivo in relevant immunocompetent mouse tumor models to better elucidate any adverse or unexpected effect.

## 1. Introduction

Tumors exhibit a complex stroma where several cellular and matrix components play an important role in sustaining tumor cell growth and survival. Immune cells such as T, B, macrophages, and dendritic cells interact with each other and with tumor cells in a paracrine manner through soluble factors such as cytokines and chemokines [1], by communicating through extracellular vesicles [2], or by direct contact cell-to-cell through receptor–ligand cognates [3]. Other cells such as fibroblasts contribute to the intricate tumor microenvironment (TME) by supporting not only tumor cell growth and immune response suppression through different soluble factors [4,5,6] but also by secreting extracellular matrix components, e.g., collagens and mucin, that affect tumor stiffness and interstitial pressure, a documented characteristic of desmoplastic tumors [4,7]. An anti-inflammatory or even an immune-deserted TME is described for most of the cancer types, hampering the efficacy of immunotherapeutic interventions such as immune checkpoint inhibitors. Factors such as TGF-β1 [8], IL-10 [9], and IL-35 [10] secreted into the TME directly inhibit cytotoxic T cells and, thus, promote an immune-suppressed TME, while others, such as CCL2, create a physical barrier that prevents immune cell infiltration; CCL2 binds its receptor on pro-tumorigenic macrophages (M2) inhibiting cytotoxic T lymphocyte infiltration leading to an immune-desert TME [11]. Tumor cells can also modulate their microenvironment expressing various immune checkpoint molecules such as the programmed cell death-1 ligand (PD-L1) through which it provokes the exhaustion of T cells [12]. Within this complexity, current immunotherapeutic approaches are devoted to overcoming the highly immunosuppressed microenvironment that surrounds tumor cells by creating hotspots for an immune system attack. In this regard, nanoparticles can play an important role in the immune priming of the TME.

Metallic nanoparticles, particularly those comprising iron oxide and gold, have been thoroughly exploited in the last decades as theranostic cancer agents in preclinical and clinical settings. Nonetheless, only recently the potential of these nanosystems as adjuvants/immunomodulators in vaccinology has been suggested [13]. The large surface for molecule engraftment [14] and the direct activation or modulation of the intracellular signaling pathway [15], thereby triggering the recruitment of myeloid and lymphoid cells, support such intrinsic ability of metallic nanoparticles to foster an immune response. However, these nanosystems could induce not only a pro-inflammatory response but also an inert or even an anti-inflammatory outcome depending on various chemical-physical characteristics, e.g., size and shape [16], coating [17,18], protein corona, or metal nature [19,20,21]. Therefore, immunological profiling should be performed for every metallic nanoparticulate system to comprehend better its immunotropic behavior and, hence, its impact on the immune response.

Polyethyleneimine (PEI) has been widely used for experimental gene transfection due to its ability to escape the early endosomal compartment [22]. The high density of positive surface charges induces a rapid and massive water flux into endosomes, thus increasing the internal pressure and, therefore, membrane disruption, thus releasing the cargo into the cytoplasm and avoiding further degradation in late endosomes upon fusing with lysosomes. However, the ability above is not the only property that emphasizes the therapeutic potential of the polycation. PEI indeed potentiates immune response by direct engagement and activation of cells belonging to the mononuclear cell system (i.e., monocytes, macrophages) through the activation of cognate receptors such as TLR4 [23,24]. Consequently, a burst of pro-inflammatory cytokines and chemokines triggers further immune system activation, including T cells, dendritic cells, and B cells. Therefore, PEI has proven to be a potent adjuvant for vaccination protocol alone or combined with TLR activators [25,26,27,28]. Another important PEI property is its weak reducing potential, thus allowing the Au^3+^ reduction to Au^0^ and further nucleation into gold nanoparticles [29]. Henceforth, PEI has been used for gold nanoparticle production in one-pot synthesis, precluding scientists from laborious intermediate steps for nanoparticle capping [30]. In conclusion, PEI is an excellent polymeric candidate for cationic gold nanoparticles in a single synthesis step.

Gold nanoparticles have been thoroughly studied as a drug delivery system for RNA/DNA [31], chemotherapeutics [32,33], and bioimaging [34,35]. Moreover, the intrinsic plasmonic properties of gold nanoparticles have permitted their exploitation as photothermal and photodynamic agents [36,37]. In the presence of external photo-activating light and a photosensitizer, gold nanoparticles can release heat (photothermal therapy) into the surrounding environment or produce large amounts of reactive oxygen species (photodynamic therapy) due to the surface plasmon resonance, eventually triggering cell apoptosis. Indeed, the induction of immunogenic cell death can prime the tumor site for immune response as tumor cell apoptosis can release large amounts of tumor-associated antigens accompanied by damage-associated molecular patterns (DAMP), such as heat-shock proteins, that can initiate local immune response activation [38,39]. These effects have prompted innovative therapeutic approaches where PTT/PDT are combined with immunotherapeutic approaches, e.g., immune checkpoint inhibitors, to boost an efficient anti-tumor immune response otherwise inefficient without the PTT/PDT priming [40,41,42]. However, gold nanoparticles can exhibit intrinsic immunomodulatory properties independent of any other features [43], which entices the study of the immunomodulatory features of all gold nanoparticles beyond the intended therapeutic purposes.

Despite the thorough reports on how gold nanoparticles [44], PEI [45], or coating iron oxide nanoparticles [23] affect immune response at several levels, there is still a gap on how the PEI-coated AuNP (hereafter named AuPEI) nanocomposites influence the tumor immunological microenvironment. We, therefore, sought to assess the immunomodulatory ability of AuPEI nanocomposites in a murine triple-negative breast tumor model based on mouse mammary cancer cell line MET-1, known for its well-infiltrated microenvironment. Contrarily to what we expected, we found that AuPEI nanocomposites shifted the immunological tumor microenvironment toward an apparent anti-inflammatory phenotype. Altogether, this finding reveals that nanoparticles can behave differently depending on the microenvironment.

## 2. Material and Methods

### 2.1. Cell Culture

Murine macrophage-like cells RAW264.7 (ATCC, TIB-71) and the MET-1 cell line [46] derived from MMTV-PyMT transgenic mouse mammary gland carcinoma, kindly obtained from Robert Cardiff (University of California, Davis), were cultured in DMEM supplemented with 10% fetal bovine serum (FBS), penicillin/streptomycin, L-glucose, and sodium pyruvate.

### 2.2. AuPEI Nanocomposite Synthesis

We followed a previously published protocol for polyethyleneimine (PEI)–AuNP nanocomposite (hereafter named AuPEI nanocomposite) synthesis [47]. Briefly, the one-pot synthesis of AuPEI was carried out by the mixture of aqueous (0.5 mg/mL, Au^3+^) HAuCl_4_·3H_2_O (G4022, Sigma-Aldrich, St. Louis, MO, USA) solution with an aqueous solution of 25 kDa PEI (40872-7, Sigma-Aldrich) at different concentrations ([PEI]: [Au] ratio of 2.5 corresponds to AuPEI-2.5, and ratio of 5 corresponds to AuPEI-5). Visual color inspection and absorbance profiling monitored the reaction evolution in the 350–800 nm wavelength range with a Multimode Plate Reader (EnSpire, Perkin-Elmer, Waltham, MA, USA). After 48 h, the resulted product was washed three times with distilled H_2_O (ultracentrifugation, 10,000× *g*) and dialyzed against distilled H_2_O using a 50 kDa cut-off membrane to remove polymer excess.

Samples were analyzed for hydrodynamic diameter distribution by dynamic light scattering (DLS). DLS measurements were carried out at 25 °C on a Zeta Sizer Nano ZS (Malvern Instruments, Malvern, UK) equipped with a 5.0 mW He-Ne laser operating at 632.8 nm and an Avalanche photodiode detector.

### 2.3. Cell Culture and Cytotoxicity Assay

RAW264.7 and MET-1 cells were cultured in complete DMEM supplemented with 10% fetal bovine serum (FBS), L-glutamine, and penicillin/streptomycin. Subconfluent cells were incubated for 24 h with increasing concentrations of AuPEI in complete DMEM. For the last 4 h, Presto Blue Cell Viability reagent was added (1/10 final dilution; Presto Blue Cell Viability Reagent, A13261; Invitrogen, Waltham, MA, USA). Fluorescence was measured with a 535 nm excitation filter and a 615 nm emission filter. Fluorescence of complete DMEM incubated with Presto Blue was used as background control. Cell viability was calculated according to the formula:

(1) $$\mathrm{Viability}\;\left(\%\right)=\frac{\left[\mathrm{AuPEI}\;\mathrm{treated}\;\mathrm{cell}\;\mathrm{fluorescence}\right]-\left[\mathrm{medium}\;\mathrm{fluorescence}\right]}{\left[\mathrm{untreated}\;\mathrm{cell}\;\mathrm{fluorescence}\right]-\left[\mathrm{medium}\;\mathrm{fluorescence}\right]}\;\times \;100$$

### 2.4. Cryo-TEM Imaging

AuPEI were characterized by cryogenic transmission electron microscopy (cryo-TEM) as published elsewhere [48]. A 4 µL droplet of particle aqueous solution was deposited on a Quantifoil^®^ (Quantifoil Micro Tools GmbH, Großlöbichau, Germany) holey carbon grid. The excess of liquid on the grid was absorbed with a filter paper, and the grid was quench-frozen quickly in liquid ethane to form a thin vitreous ice film. Once placed in a Gatan 626 cryo-holder cooled with liquid nitrogen, the samples were transferred to the microscope and observed at low temperature (−180 °C). Cryo-TEM images were recorded with a 2 k × 2 k Gatan Ultrascan 1000 CCD camera (Gatan, Pleasanton, CA, USA), using a LaB_6_ JEOL JEM2100 (JEOL, Tokyo, Japan) cryo-microscope operating at 200kV. Images were taken with the JEOL low-dose system (Minimum Dose System, MDS) to protect the thin ice film from any irradiation before imaging and reduce the irradiation during the image capture (IMPMC, Sorbonne Université, 4 place Jussieu, Paris 75005, France).

### 2.5. Cell TEM Imaging

After incubation with AuPEI for 24 h, cells were harvested, washed 3× with PBS 1×, and resuspended in fixing buffer (0.1 M sodium cacodylate, 2.5% glutaraldehyde). Cell suspensions were mixed gently for 1 h at 4 °C. After being washed twice, cells were resuspended in 0.1 M sodium cacodylate buffer until the sample preparation. Samples were then contrasted with oolong tea extract (OTE) 0.5% in cacodylate buffer, post-fixed with 1% osmium tetroxide containing 1.5% potassium cyanoferrate, gradually dehydrated in ethanol (30% to 100%), and substituted gradually in a mix of ethanol-Epon and embedded in Epon (Delta Microscopie–Labège, France). Thin sections (70 nm) were collected onto 200 mesh copper grids and counterstained with lead citrate. Grids were examined with Hitachi HT7700 electron microscope operated at 80 kV (Elexience–Verrieres Le Buisson, France), and images were acquired with a charge-coupled device camera (AMT) at MIMA2 MET–GABI, INRAE, Agroparistech, 78352 Jouy-en-Josas, France.

### 2.6. Macrophage Activation

A total of 10^5^/mL murine macrophage cells, RAW264.7 (ATCC, TIB-71), were cultured with 5 µg/mL AuPEI or lipopolysaccharide (LPS) (L4005, Sigma-Aldrich) for 24 h in 5% CO_2_ at 37 °C. Then, the supernatant was collected and kept at ~80 °C until ELISA procedures.

### 2.7. ELISA

Levels of murine secreted IL-10 (DuoSet ELISA DY417-05, R&D Systems, Minneapolis, MN, USA), IL-6, CCL5, MMP2, SerpinE1, TIMP-2, and TNFα (DuoSet ELISA DY410-05, BD Biosciences, Franklin Lakes, NJ, USA) were quantified according to instructions. Absorbance was read with a Multimode Plate Reader (EnSpire, Perkin-Elmer). Triplicate samples were analyzed.

### 2.8. In Vivo Experiment

All animal experimentations were carried out in agreement with the institute Ethical Committee from Université Paris Cité. MET-1 allograft was established by subcutaneous injection of 1.5 × 10^6^ cells/50 µL PBS in the flank of C57BL/6 mice (6–8 weeks old). Tumor volume was monitored with a caliper and calculated as follows: V = (xy^2^)/2, where x is the longest and y the shortest of the perpendicular diameters.

Approximately 10 days after cell injection, MET-1 tumor-bearing mice (tumor size ≥ 5 mm, volume ~250 mm^3^) were randomized into three groups as follows: (i) control 40 µL saline solution (n = 3); (ii) intratumoral injection of 20 µg AuPEI-2.5 in 40 µL saline solution (n = 3); and (iii) intratumoral injection of 20 µg AuPEI-5 in 40 µL saline solution (n = 3). After 24 h, mice were sacrificed and tumor excised for isolation and analysis of tumor-infiltrating lymphocytes and myeloid cells.

Intratumoral infiltrating lymphocyte (TIL), tumor-associated macrophage (TAM), and dendritic cell (TADC) cytometric analysis: Immune cells in tumors were stained as described previously [49]. Each tumor was individually analyzed. Briefly, tumors were mechanically dissociated and digested for 45 min at 37 °C in RPMI 1640 with 37.5 µg/mL Liberase^TM^ (Roche, Basel, Switzerland) and 8000 U/mL DNase I from bovine pancreas (Merck Millipore, Burlington, MA, USA). After being filtered (70 µm cell strainer) and centrifuged, the red blood cells were lysed with ACK buffer on the remaining pellet and subsequently filtered through a 40 µm cell strainer. The cell suspension was then rinsed in PBS and stained in 96-well round-bottom plates with a LIVE/DEAD Fixable Blue Dead Cell Stain Kit (Invitrogen) for 20 min at 4 °C. Cells were washed and stained with antibodies (Ab) against surface proteins at a 10 µg/mL concentration for 20 min at 4 °C. After surface staining, cells were fixed with BD Fixation and Permeabilization Solution for 20 min at 4 °C. After washing in PBS, cells were resuspended in PBS 2% FBS and analyzed with a BDFortessa flow cytometer (BD Bioscience). Data were analyzed by FlowJo software.

The anti-mouse antibodies used were the following (Table 1):

### 2.9. Statistical Analysis

Data were analyzed and depicted with GraphPad Prism and FlowJo software. Data are presented as mean ± SEM, and the number of samples and independent experiments are stated in each figure caption for precision. Data were analyzed with a one-tailed Mann–Whitney test under the assumption of non-Gaussian distribution (nonparametric test), with a 95% confidence, and * *p* < 0.05.

## 3. Results

### 3.1. AuPEI Nanocomposite Synthesis and Characterization

**Figure 1:**
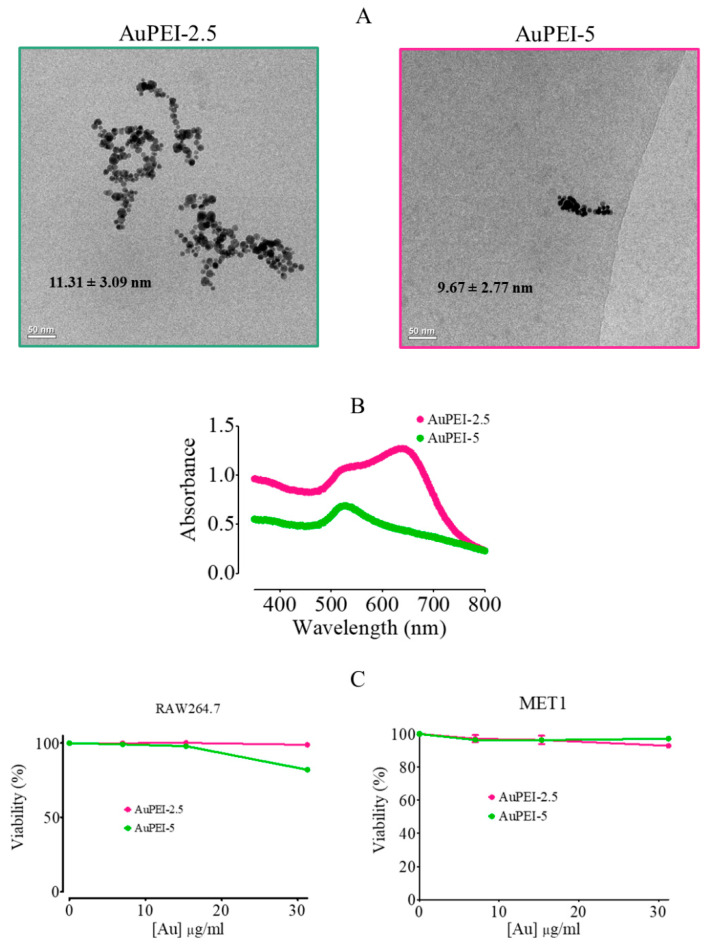
AuPEI nanocomposite characterization and toxicity. (**A**) Cryo-TEM imaging of AuPEI nanocomposites at 30 days. (**B**) One-year-old AuPEI nanocomposite absorbance profile showing the primary and secondary plasmon resonance band. (**C**) Viability of RAW264.7 and MET-1 cell lines after incubation with different concentrations of AuPEI nanocomposites.

To obtain the AuPEI nanocomposites, we followed a one-pot synthesis method [47] where 0.5 mg/mL Au^3+^ solution (prepared from HAuCl_4_·3H_2_O salt) was mixed thoroughly with polyethyleneimine (PEI) for a final [PEI]: [Au] ratio of 2.5 and 5 (Figure 1A). The as-synthesized nanocomposites were directly dispersed in milliQ water. This one-pot synthesis is based on the weak reducing potential of amine residues present in the polymer. AuPEI nanocomposites produced at [PEI]:[Au] ratios of 2.5 (AuPEI-2.5) and 5 (AuPEI-5) showed a gold nanoparticle core diameter of 11.3 and 9.7 nm, respectively, as measured by cryogenic transmission electron microscopy (cryo-TEM) (Figure 1A), and arrangement into clusters as described in detail in [50]. One-year-old AuPEI exhibited the canonical localized surface plasmon resonance (LSPR) band around 525 nm corresponding to the transversal mode; nonetheless, AuPEI-2.5 also displayed a secondary longitudinal LSPR band, suggesting a plasmon coupling phenomenon most likely due to the closeness of nanoparticles in the fractal-like clusters (Figure 1B). Importantly, we did not detect significant cell toxicity at lower AuPEI concentrations (<30 µg/mL) (Figure 1C), both in the mouse RAW264.7 macrophage cell line and in MET-1 tumor cells.

### 3.2. AuPEI Nanocomposites Activate Murine Macrophage Cell Line, RAW264.7

**Figure 2:**
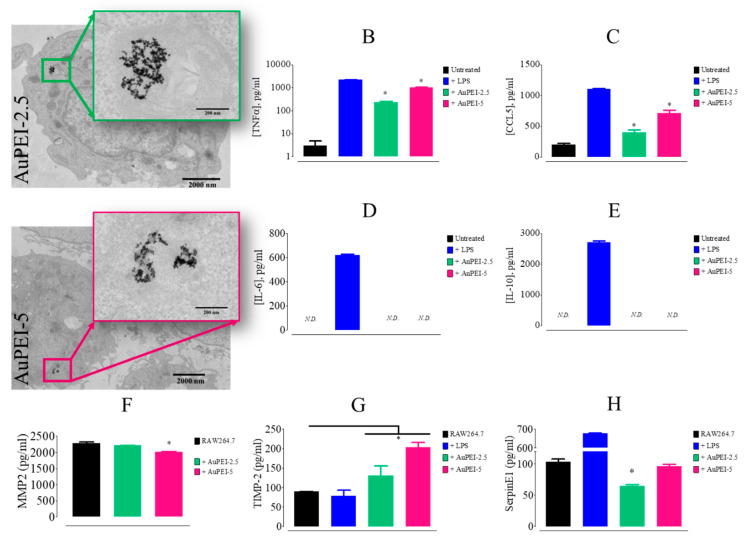
AuPEI nanocomposite effects on murine macrophage activation status. (**A**) TEM imaging of RAW264.7 cells incubated with 5 µg/mL AuPEI nanocomposites for 24 h. Supernatant level of secreted (**B**) TNFα, (**C**) CCL5, (**D**) IL-10, (**E**) IL-6, (**F**) MMP2, (**G**) TIMP-2, and (**H**) SerpinE1. (n = 6; * *p* < 0.05 Mann–Whitney test, 95% confidence).

Based on previously published reports on PEI immunomodulatory intrinsic ability [23,26], we sought to assess whether AuPEI nanocomposites affect macrophage activation status in vitro. After 24 h of treatment, AuPEI nanocomposites (5 µg/mL) were arranged in endosome-confined clusters in RAW264.7 cells (Figure 2A). Concomitantly, murine macrophages RAW264.7 secreted a high level of pro-inflammatory cytokine TNFα (Figure 2B) and chemotactic chemokine CCL5, known for its pivotal role in recruiting leukocytes to inflamed tissues (Figure 2C). However, we did not detect pro-inflammatory IL-6 or immune suppressive IL-10 cytokines in the supernatant after 24 h of treatment (Figure 2D,E). Noteworthy, murine macrophages exposed to AuPEI-5 and AuPEI-2.5 exhibited a significant increase in metalloproteinase inhibitor TIMP-2 (Figure 2G), together with a slight decrease in metalloproteinase MMP2 secretion (Figure 2F) suggesting that the macrophage migratory and invasion behavior could be affected in a relevant tissue environment [51,52]. SerpinE-1, another metalloproteinase inhibitor, either slightly decreased (AuPEI-2.5) or was unchanged (AuPEI-5) (Figure 2G).

Overall, our results indicate that AuPEI exposure for 24 h has significant effects on RAW macrophage secretion with important upregulation of inflammatory cytokines such as TNFα. Metalloproteinase inhibitor TIMP-2 was also affected in a significant manner. However, the use of macrophage-like cell lines, independently of a relevant tissue-specific or disease-specific environment, is oversimplified to elucidate the multifactorial effect of nanocomposites.

We thus switched to a more specific and well-known immune-infiltrated tumoral environment in which particles were injected intratumorally. Intratumoral injection is particularly relevant for nanoparticles such as AuNPs that can be activated at distance for photothermal therapy or radiosensitization in clinical practice. They can be injected transcutaneously or by endoscopy in order to shrink the tumor before resection as an adjuvant treatment. Intratumoral injection is the best option to maximize the tumor concentration of nanoparticles, minimize their release in the circulation and translocation to healthy tissue and diminish side effects. Moreover, nanoparticles can eventually exert immunomodulatory effects on their own directly in the context of the tumoral microenvironment which can prime the TME before irradiation. For all these reasons, we aimed to characterize the effects of AuPEI directly in the TME of the MET-1 syngeneic mice model of triple-negative breast cancer.

### 3.3. Local Tumor Modulation of Myeloid Population Induced by AuPEI Nanocomposites in MET-1 Breast Cancer Model in Immunocompetent Mise

Myeloid cells respond to environmental cues within tissues such as damaged cells, activated lymphocytes, xenobiotics, and nanoparticles to differentiate into distinct functional phenotypes [53,54]. Patrolling tissues through vasculature for any damage or danger highly depends on monocyte subsets. Of them, inflammatory monocytes play an important role as these cells express toll-like receptors (TLRs) and scavenger receptors that mediate the recognition of pathogen-associated molecular patterns and damage-associated molecular patterns. As a consequence, inflammatory monocytes infiltrate tissues where they can differentiate into macrophages and release effector molecules such as cytokines, myeloperoxidase, and superoxide, thereby initiating inflammation [55,56].

M1 macrophage phenotype produces high levels of pro-inflammatory cytokines in order to mediate resistance to pathogens, microbicidal properties, and promotion of Th1 responses. In the context of cancer, M1 macrophages can initiate and sustain inflammation, which can activate the immune response against cancer cells. In contrast, M2 macrophages generally involved in tissue remodeling and phagocytic activity can regulate immunity and participate in tumor promotion by immunosuppression. Reprogramming of protumoral M2 macrophages into inflammatory M1 subtypes has been proposed as part of anti-tumor therapy mediated by iron oxide nanoparticles [57].

**Figure 3:**
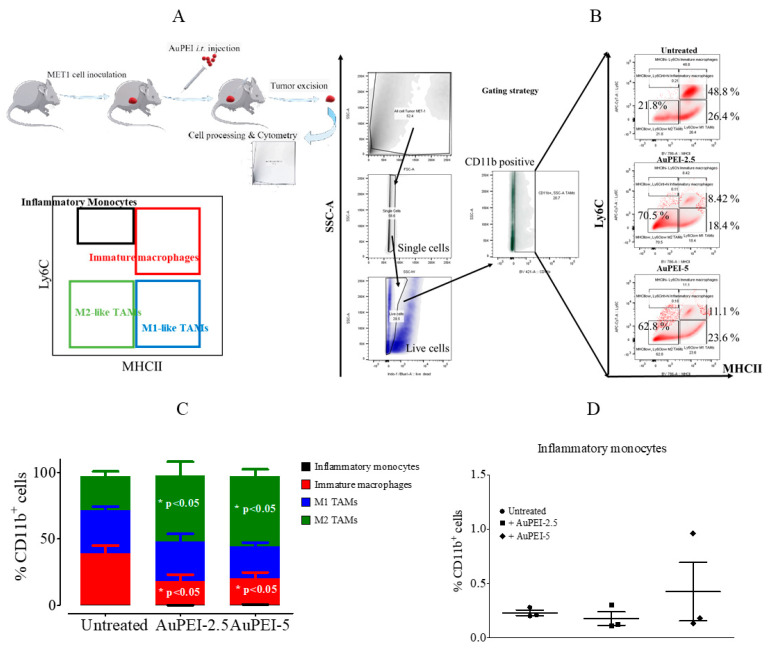
MET-1-infiltrating tumor-associated macrophage (TAM) distribution upon AuPEI nanocomposite i.t. injection. MET-1-bearing mice were injected i.t. with 40 µL of 0.5 mg/mL AuPEI nanocomposites and euthanized 24 h after. (**A**) Experimental procedure and gating strategy to differentiate TAMs within the tumor cell population. Inflammatory monocytes defined as *CD11b^+^ MHCII^−^ Ly6C^hi^*; immature macrophages defined as *CD11b^+^ MHCII^hi^ Ly6C^hi^*; M1-like tumor-associated macrophages defined as *CD11b^+^ MHCII^hi^* *Ly6C^−^*; and M2-like tumor-associated macrophages defined as *CD11b^+^ MHCII^−^ Ly6C^−^*. (**B**) A representative dot-plot shows the percentage of different TAM populations upon AuNP nanocomposite injection and (**C**) summary of TAM distribution. (**D**) Distribution of inflammatory monocytes within tumor nest. (Results are shown as mean ± SEM of n = 3 per condition; * *p* < 0.05 Mann–Whitney test, 95% confidence).

To analyze the impact that AuPEI nanocomposites might exert on the TME, we injected intratumorally AuPEI nanocomposites (20 µg/40 µL, Au) in a mouse mammary tumor MET-1 (~10 days after tumor cell injection, ~250 mm^3^), a known immunogenic tumor type. A different infiltrating immune cell subpopulation was then analyzed by cytometry 24 h after injection for each tumor/mouse individually according to a gating strategy published elsewhere [58] (Figure 3A,B). As in the in vitro study, two different AuPEI nanocomposites were assessed, i.e., AuPEI-2.5 and AuPEI-5, in comparison to the vehicle injection. In the gating strategy represented in Figure 3B, first, the singlet and live cell populations were selected, and second, we focused our analysis on the myeloid subset *CD11b^+^*. From the *CD11b^+^* population, we can distinguish four subsets: (1) inflammatory monocytes defined as *CD11b^+^ MHC (Major histocompatibility complex II)II^−^ Ly6C^hi^*; (2) immature macrophages defined as *CD11b^+^ MHCII^hi^ Ly6C^hi^*; (3) M1-like tumor-associated macrophages defined as *CD11b^+^MHCII^hi^ Ly6C^−^*; and (4) M2-like tumor-associated macrophages defined as *CD11b^+^ MHCII^−^ Ly6C^−^*. These subsets describe the evolution of infiltrating monocytes and macrophages once inside the tumor microenvironment.

As shown in Figure 3B,C, 48.8% of the *CD11b^+^* population exhibits an immature macrophage phenotype, while M1-like and M2-like macrophage subpopulations represent 26.4% and 21.8%, respectively. Strikingly, the AuPEI nanocomposites significantly decreased the percentage of tumor-infiltrating immature macrophages (*CD11b^+^ MHCII^hi^ Ly6C^hi^*) from 48.8% down to 8.42% (AuPEI-2.5) and 11.1% (AuPEI-5) (Figure 3B,C). Moreover, it appeared that the decrease in immature macrophages concurred with a significant increment in the percentage of M2-like tumor-associated macrophages (M2-like TAMs) from 21.8% up to 70.5% (AuPEI-2.5) and 62.8% (AuPEI-5), suggesting a conversion of the immature macrophages into M2-like macrophages over treatment with both AuPEI2.5 and AuPEI5 but not into M1-like macrophages (Figure 3C). Furthermore, we did not detect a significant variation in inflammatory monocyte proportion (Figure 3D). Altogether, AuPEI nanocomposites seem to favor an immunosuppressive tumor microenvironment by increasing the M2-like: M1-like TAM ratio.

**Figure 4:**
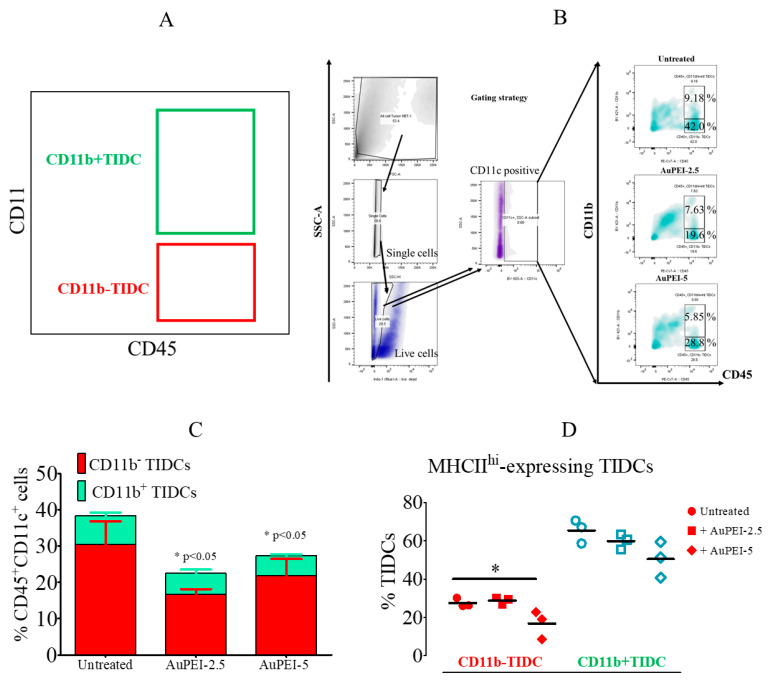
MET-1 tumor-infiltrating DC distribution upon AuPEI nanocomposite i.t. injection. MET-1-bearing mice were injected i.t. with 0.5 mg/mL Au (40 µL) AuPEI nanocomposites and euthanized 24 h after. (**A**) Gating strategy to differentiate TIDCs within the tumor cell population. (**B**) Representative dot-plot showing percentage of different TIDC populations upon AuNP nanocomposite injection, and (**C**) summary of the distribution of TIDC population. (**D**) Activation status of TIDCs as measured by MHCII expression. (Results are shown as mean ± SEM of n = 3 per condition; * *p* < 0.05 Mann–Whitney test, 95% confidence).

Among all myeloid cells, dendritic cells (DCs) are the most potent at inducing an efficient adaptive immune response as these cells process and present foreign antigens, e.g., tumor-associated antigens, to lymphocytes (B and T cells) in the context of MHC. Therefore, the presence of DCs in a TME is imperative for a proper anti-tumor immune response [59]. Dendritic cells normally represent a small fraction (1–5%) of the myeloid infiltrate in non-lymphoid tissues [60], and, although CD11c has been recently associated with a fraction of macrophages [61], CD11c remains still a *bona fide* DC marker. We thus analyzed the effect of AuPEI nanocomposites on tumor-infiltrating dendritic cells (TIDCs) based on the *CD11c^+^CD45^+^* phenotype, which, according to the gating strategy, merely represents 4.44% of the whole cell population in untreated tumors. In untreated tumors, the *CD11c^+^CD45^+^ CD11b^hi^* subset merely represents 0.79% of the whole cell infiltrate, indicating that the incidence of *CD11b* in the prior analysis of *CD11b^+^* monocyte/macrophage gating is negligible. However, among the TIDC population, the *CD11b^h^*^i^ subset is often associated with poor tumor progression prognosis [62,63] or tolerogenic scenarios [64] and can have a great influence on the behavior of TIDCs (Figure 4A). Noteworthy, AuPEI nanocomposites lowered the proportion of both *CD11b^−/low^*- and *CD11b^hi^*-TIDC subsets (Figure 4B,C). Moreover, AuPEI-5 nanocomposites appeared to decrease the proportion of *MHCII^hi^*-expressing cells within the *CD11b^−^* and *CD11b^+^* TIDC subsets (Figure 4D).

### 3.4. Local Tumor Modulation of Lymphoid Population Induced by AuPEI Nanocomposites

**Figure 5:**
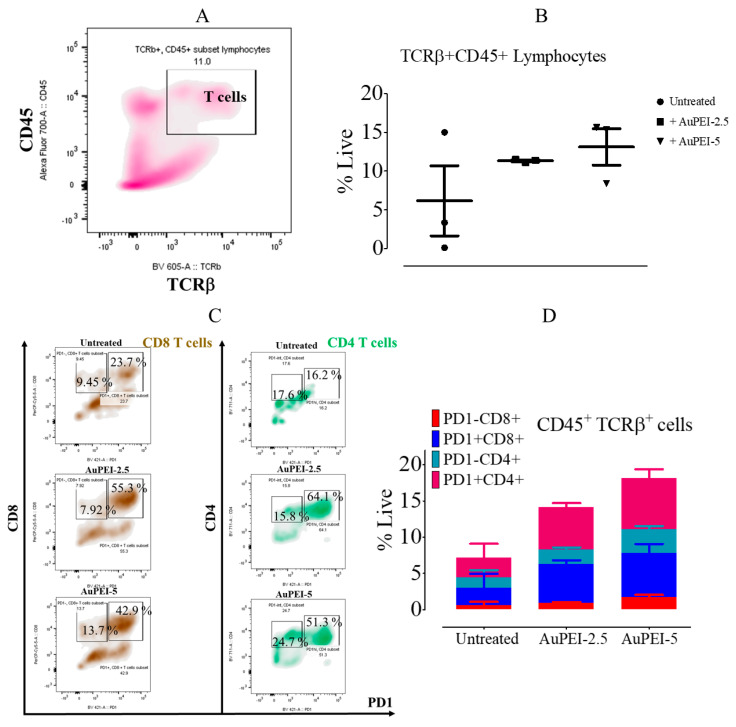
MET-1 tumor-infiltrating lymphocyte (TIL) distribution upon AuPEI nanocomposite i.t. injection. MET-1-bearing mice were injected i.t. with 0.5 mg/mL Au (40 µL) AuPEI nanocomposites and euthanized 24 h after. (**A**) Gating strategy to differentiate TILs within the tumor cell population. (**B**) Percentage of total TILs in tumors. (**C**) Representative dot-plot showing expression of PD-1 by CD4 and CD8 T cells, and (**D**) summary of the distribution of CD4 and CD8 T cells according to PD-1 expression. (Results are shown as mean ±SEM of n = 3 per condition).

Since we observed a profound impact on MET-1 tumor-infiltrating myeloid cell populations upon AuPEI nanocomposite treatment in vivo, we sought to determine whether this phenomenon concurs with a change in tumor-infiltrating lymphocyte (TIL) subsets (Figure 5A). T cell subsets are perfectly distinguished by the co-expression of the receptor-type protein tyrosine phosphatase, CD45 [65], and the β chain of the T-cell receptor, TCRβ [66]. Both markers are among the most abundant proteins within the T-cell plasma membrane and are required for T-cell receptor signaling. Gating on live cells, we indeed noticed a trend in TIL (*TCRβ^+^CD45^+^*) percentage increase in all cases; TIL percentages varied from 6.17 ± 7.8% up to 11.33 ± 0.20% (AuPEI-2.5) and 13.11 ± 4.06% (AuPEI-5) (Figure 5B). Nonetheless, this increase in TIL was not homogeneous as the ratio of CD4/CD8 also increased from ~1 (untreated) up to 1.26 (AuPEI-2.5) and 1.34 (AuPEI-5).

It is known the role of the CD28 family member, PD-1, in cancer progression through a plethora of immunosuppressive signals it cues upon engagement with PD-L1/2 ligands [67]. Indeed, PD-1 belongs to an extensive family of regulatory molecules called checkpoints that regulates T-cell activation in negative feedback and is currently one of the most prominent targets in cancer immunotherapy [68]. In our study, PD-1^+^ CD4 and CD8 T-cell subsets showed significant increment as compared to PD-1^−^ counterparts (Figure 5C,D). While PD-1^−^ CD8^+^ cell percentage within the TIL population decreased from 9.45% to 7.92% (AuPEI-2.5) and increased to 13.7% (AuPEI-5), PD-1^+^ CD8^+^ cell percentage increased from 33.25% to 63.22% (AuPEI-2.5) and 56.6% (AuPEI-5); as such, the PD-1^+^/PD-1^−^ ratio in CD8^+^ T cells increased from 2.5 (untreated) to 6.98 (AuPEI-2.5-treated) and 3.13 (AuPEI-5-treated). A similar behavior was observed in the CD4^+^ T-cell compartment, where the PD-1^+^/PD-1^−^ ratio increased from 0.92 (untreated) to 4.06 (AuPEI-2.5-treated) and 2.08 (AuPEI-5-treated) (Figure 5C,D). The increment in PD-1^+^ T cells correlates with the apparent immunosuppressor microenvironment induced by AuPEI nanocomposites and might contribute to this phenomenon.

**Figure 6:**
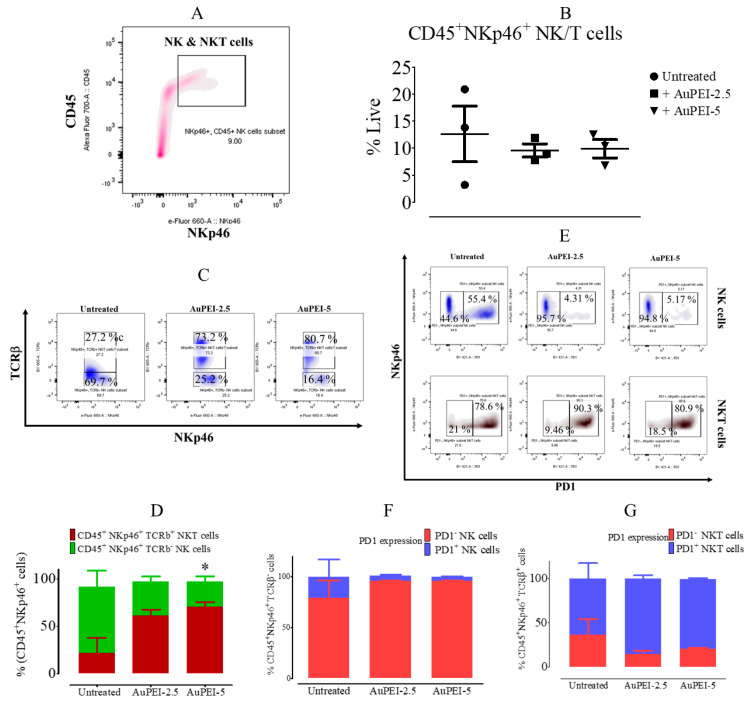
MET-1 tumor-infiltrating NK cell distribution upon AuPEI nanocomposite i.t. injection. MET-1-bearing mice were injected i.t. with 0.5 mg/mL Au (40 µL) AuPEI nanocomposites and euthanized 24 h after. (**A**) Gating strategy to differentiate NK/NKT cells within the tumor cell population. NK/NKT cells, *CD45^+^NKp46^+^*; NK, *CD45^+^NKp46^+^TCRβ^−^*; and NKT cells, *CD45^+^NKp46^+^TCRβ^+^*. (**B**) Percentage of total NK/NKT cell population in tumors. (**C**) Representative dot-plot and (**D**) summary of NK and NKT cell distribution within the total NK/NKT cell population. (**E**) Representative dot-plots show PD-1 expression by NK cells (upper) and NKT cells (lower); and a summary of PD-1 expression by (**F**) NK cells and (**G**) NKT cells. (Results are shown as mean ± SEM of n = 3 per condition; * *p* < 0.05 Mann–Whitney test, 95% confidence).

Natural killer cells are also part of the lymphocyte family, but unlike T and B cells, NK cells are part of the innate immune system providing first-line defense against viruses, bacteria, and cancer [69,70]. In addition, NKT cells share T and NK cell features, mostly associated with the recognition of self and non-self lipids and glycoproteins bound to the non-polymorphic CD1d molecule [71]. NKp46 is a 46 kDa membrane glycoprotein that mediates the specific activation of the NK cell lineage, including NKT cells, thus participating in unrestricted natural cytotoxicity [72,73]. Therefore, NKp46 is a *bona fide* marker for NK and NKT cells. We thus analyzed the NK lineage within the lymphocytic population infiltrating the MET-1 tumors by co-staining CD45, a leukocyte marker highly expressed in lymphocytes, and the NK-lineage-specific marker, NKp46. To distinguish NK (*CD45^+^NKp46^+^TCRβ^−^*) from NKT (*CD45^+^NKp46^+^TCRβ^+^*) cells, we also co-stained for TCRβ as this receptor specifically identifies T cells; thus, co-staining of TCRβ and NKp46 identifies NKT cells. Although the total NK/NKT subpopulation (*CD45^+^NKp46^+^*) did not vary significantly (untreated, 12.63 ± 8.91%, vs. AuPEI-2.5-treated, 9.56 ± 2.11%, and AuPEI-5-treated, 9.88 ± 2.91%; Figure 6A,B), AuPEI nanocomposites changed the NK/NKT balance favoring NKT (*CD45^+^NKp46^+^TCRβ^+^*) cell proportion to the detriment of the NK (*CD45^+^NKp46^+^TCRβ^−^*) cell subset (untreated NK/NKT ratio ~2.56 vs. AuPEI-2.5-treated NK/NKT ratio ~0.34 and AuPEI-5-treated NK/NKT ratio ~0.20; Figure 6C,D).

**Scheme 1:**
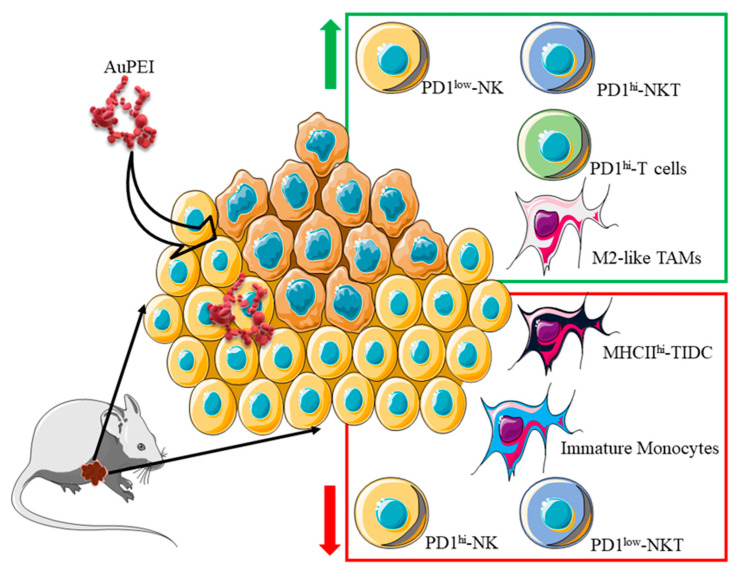
General AuPEI nanocomposite effect on MET-1 tumor-immune microenvironment. Intratumoral injection of AuPEI nanocomposites increased the proportion of PD-1^hi^-NKT, PD-1^hi^ T, PD-1^low^ NK cells, and M2-like TAMs. Concomitantly, AuPEI nanocomposites diminished the proportion of *MHCII^+^* TIDCs and immature monocytes. Altogether, the nanocomposites trigger a pro-tumorigenic landscape in the MET-1 tumor nest.

Unlike the T-cell compartment, PD-1 has a diverse behavior between NK and NKT cells. That is, the PD-1^+^ NK cell subset decreased in proportion to PD-1^−^ NK cells (Figure 6E upper and Figure 6F) while in the NKT subpopulation occurred the opposite balance shift (Figure 6E lower and Figure 6G). We can then summarize the collective effect of AuPEI nanocomposites on reprogramming murine mammary tumor MET-1 microenvironment for infiltrating immune cells as shown in Scheme 1. As such, AuPEI seem to induce a heavy infiltration of T cells that exhibit a rather exhausted phenotype (PD-1^+^) and increment in the proportion of the anti-inflammatory M2-like macrophages.

## 4. Discussion

The use of inorganic nanoparticles for cancer treatment has grown enormously in the last decades due to the versatility of these nanosystems. Nonetheless, critical concern is raised as their utilization extended into in vivo models, i.e., whether the nanoparticles interact with the immune system and the nature of this interaction. In the context of cancer, this putative immunotropic behavior of nanoparticles is pivotal to comprehend better whether nanoparticle application changes tumor immunological microenvironment in favor of or to the detriment of an efficient anti-tumor immune response. It is indeed such interaction and its nature that could potentially drive the decision making on the best combination with current and future immunotherapeutics in order to improve anticancer response. We found that PEI-coated gold nanoparticles (AuPEI) triggered TNFα and CCL5 release by murine macrophages RAW264.7 in vitro. TNFα is an intriguing cytokine formerly thought to be pro-inflammatory, hence anti-tumoral cytokine. Nonetheless, TNFα has also been associated with tumor progression lately [74], and this dual activity seems to depend on its spatiotemporal availability [75] and pleiotropic effects on the tumor microenvironment, leading even to impairment in cytotoxic CD8^+^ T cells within the tumor niche [76]. Likewise, CCL5, which is mainly secreted by T cells, has been linked to a pro-tumorigenic effect as its interaction with the cognate receptor CCR5 triggers tumor cell proliferation and progression into a more aggressive phenotype [77,78]. We, therefore, interrogated a highly immunogenic tumor microenvironment, i.e., MET-1, for the immunological phenotype upon AuPEI intratumoral injection to better comprehend the global effect in vivo.

Our results support that AuPEI induces a putative pro-tumor microenvironment by changing the balance within both myeloid and lymphoid populations. Tumor development (growth, progression, and metastasis) highly depends on the phenotype of the tumor-associated macrophages (TAMs) present in the tumor environment [79]. The accepted paradigm refers to M1 as anti-tumorigenic and M2 as pro-tumorigenic and both types of the landscape tumor microenvironment as their balance varies. As such, TAMs are considered primordial for aggressive tumor behavior [80]. We found that within the TAM population in MET-1 tumors, as dissected according to procedures published elsewhere [58,81], AuPEI increased the M2-like subpopulation, more likely due to the differentiation of immature macrophages rather than the M1-like TAM reconversion, as the proportion of M1-like TAM does not change significantly. Henceforth, a precise balance favoring a pro-tumorigenic environment (M2/M1) agreed with the increase in TNFα and CCL5 in RAW264.7 cells. However, while AuPEI favors the balanced M2/M1, they appear to diminish the immunosuppressive tumor-infiltrating DCs (*CD11b^+^* TIDCs) [62,82], although the proportion of *MHC-II^+^* TIDCs also decreased. The tumor-infiltrating *CD11b^+^* *CD11c^+^* myeloid cells, however, have also been associated with a totally opposite function in a murine mammary carcinoma model. Infiltrating *CD11b^+^CD11c^+^* myeloid cells have proven to have great potential in mediating cell death of mammary carcinoma of HER-2/neu transgenic mice. These effects appeared to depend on inducible nitric oxide synthase [83]. Therefore, the decrease in the proportion of *CD11b^+^* *CD11c^+^* myeloid cells might as well facilitate MET-1 tumor cell survival and progression independently of the increase in the tumor immunosuppressive microenvironment. Further studies should be performed on the functions of these different myeloid cells as per their cytokine/chemokine profile and suppressor capacity in vitro.

In our MET-1-based in vivo model, it appears that there is an increment in tumor-infiltrating lymphocytes (untreated, 6.17%, vs. AuPEI-2.5-treated, 11.33%, and AuPEI-5-treated, 13,11%). It is now generally accepted that a tumor contains a great population of infiltrating lymphocytes with broad specificity for self or mutated tumoral antigens but inactive against tumor cells. More important, sometimes such lymphocytes exhibit markers for clonal expansion. Therefore, a huge effort has been focused on reactivating such lymphocytic populations. Because of the relatively short time elapsed from the AuPEI intratumoral injection and the increase in TILs, it is unlikely that such lymphocytic number explosion arises from a clonal expansion. It rather indicates an influx from circulating lymphocytes. Noteworthy, the time elapsed from the nanoparticle intratumoral injection and the analysis of tumor-infiltrating immune cells can affect the final results as it is rather a dynamic process. For instance, Nicolás-Boluda A et al. found that gold-coated iron oxide nanoflowers induced an increase in the CD4/CD8 ratio after 24 h, like in our system. However, this ratio significantly decreased after 12 days concomitantly with a drop in the CD4 T cell population in MET-1 tumors [84]. Thus, a long-term analysis should be performed in the future with the AuPEI.

Although CD4^+^ and CD8^+^ T-cell proportion was greatly augmented within the MET-1 tumors, most of these cells express the checkpoint molecule PD-1, likely attenuating T-cell activation [67]. From our experiments, it is not clear whether the T cells infiltrating the tumor already exhibited an exhausted phenotype (PD-1 expression) or gained such phenotype once within the tumor. Nonetheless, the high expression of PD-1 might be therapeutically useful in an anti-PD-1 therapeutic approach. That is, we can treat tumors with AuPEI, which leads to an increment in PD-1^+^ T cells, combined with an anti-PD-1 or -PD-1L antibody which, in turn, can reactivate these T cells to react against tumor cells. Thus, AuPEI might function as an attractor for T cells into the tumor where a checkpoint inhibitor could reactivate them.

It is well known the anti-tumoral activity of NK cells as part of the innate lymphoid cells [85,86]. Moreover, NKT cells are known to bridge innate and adaptive immune responses. In particular, NKT cells play a crucial role in tumor immunity based on the large amount of IFN-γ these cells produce upon engagement of CD1d-presented lipids [87,88]. Curiously, it is described that in anti-PD-1-resistant tumor models, the activation of NKT cells overcomes such resistance by reinvigorating exhaustive CD8^+^ T cells [89]. Therefore, increasing the NKT population upon i.t. injection of AuPEI, from 27.2% to 73.2% (AuPEI-2.5) and 80.7% (AuPEI-5), can potentiate the anti-tumor immunity if an external NKT-activating factor is implemented. However, a slight rise in the PD-1-expressing NKT cell subpopulation might pose another hurdle for the proper anti-tumor immune response.

Since there is no evidence in the literature for the conversion of NK cells into NKT cells, and the fact that some NKT cells can express CD4 and CD8, we believe that the increment in the NKT cell subpopulation might arise from the high infiltrate of the lymphocytic population after AuPEI injection. Thus, reducing intratumoral NK cell and PD-1-positive NKT cell proportion might hinder the natural anti-tumoral immune response in the MET-1 tumor model (Scheme 1). However, the combination with an NKT-activating ligand could potentially overcome this phenomenon. Like T-cell subsets, the combination with an anti-PD-1 antibody might counteract the signaling cascade driven by PD-1 on NKT cells, reinforcing a putative anti-tumor activity. Altogether, the lymphoid population suffers a shift toward an immunosuppressive and, henceforth, pro-tumoral phenotype at least through the rise in PD-1^+^ T cells and a significant decrease in NK cell proportion.

It is not the first time that gold nanoparticles have shown immunosuppressive effects. For instance, gold nanoflowers synthesized with the extract of Acanthopanacis cortex hamper LPS-induced activation of RAW264.7 macrophages by reducing the expression of enzymes iNOS and COX2 in vitro [90]. Likewise, spherical silver and hexagonal gold nanoparticles inhibit NF-κB activation in LPS-activated RAW264.7 cells, thereby exerting an anti-inflammatory effect [91]. In addition, gold-organic compound complexes have been used for decades to treat inflammation, specifically arthritis rheumatoid [92]. Even spherical citrate-coated gold nanoparticles (5 nm) exhibit a complete disruption of the IL-1β pathway in THP1 cells, and this effect disappears as the gold nanoparticle diameter increases [93]. Furthermore, 21 nm gold nanoparticles seem to trigger a systemic anti-inflammatory response in mice as measured by the level of TNFα mRNA and IL-6 mRNA in the abdominal fat [43]. More recently, alkyl-terminated gold nanoparticles have proven to prevent imiquimod-induced psoriasis by inhibiting the IL-17 pathway in mice [94]. However, most of these studies have been performed in the context of inflammatory disorders, and little is known about the collective effect that gold nanoparticles have on the tumor microenvironment so far. Since gold nanoparticles are being thoroughly studied as drug delivery systems and photodynamic and photothermal therapies in cancer, it is pivotal to understand how these nanoparticles prime the TME for possible immunotherapy approaches. Here, the main implications of our findings in how AuPEI can reshape the MET-1 tumor microenvironment are that it can potentially prime tumors for a checkpoint inhibitor (anti-PD-1 or anti-PD-L1 antibody) treatment, enhancing the therapeutic efficacy of the immunotherapy.

Furthermore, the manner in which AuPEI reshape the TME might also influence the efficacy of other therapeutic interventions such as PTD/PTT where an immunogenic cell death can be induced. We indeed demonstrated that AuPEI can trigger tumor hyperthermia upon laser irradiation [47]. Hereby, we demonstrated that the intratumoral injection of AuPEI alone is capable of shifting the tumor immunological microenvironment toward an anti-inflammatory response where PD-1 signaling may prevail. In this scenario, we foresee a triple therapeutic approach: AuPEI-induced PD-1-enriched immunological tumor microenvironment; assisted immunogenic cell death induced by photothermal therapy that potentiates the presentation of tumor-associated antigens to the local and systemic immune system; and the concomitant administration of an anti-PD-1 antibody to reactivate tumor-infiltrating lymphocytes. Altogether, not only can AuPEI attract heavy infiltration of lymphocytes but also can further stimulate the immune system locally and systemically. These AuPEI potentials entice further study in multitherapeutic approaches in the future.

Moreover, the nature of nanoparticle coating can also affect the immune response as demonstrated elsewhere. As such, 20 nm spherical gold nanoparticles produced by ultrasonic spray pyrolysis but with different stabilizers (sodium citrate, poly-ethylene glycol, or polyvinyl-pyrrolidone) exhibit differential immune-modulation. While citrate-coated and PEG-coated gold nanoparticles greatly inhibit the production of pro-inflammatory cytokines by human peripheral blood mononuclear cells, e.g., IFNγ, IL-1, IL-6, IL-8, and TNFα, the PVP-coated gold nanoparticles promote an inflammatory environment by triggering the production of T_H_1 cytokines [95]. AuPEI, however, are coated with polyethyleneimine (PEI), a well-known pro-inflammatory polymer [27,45] which indicated the possibility that AuPEI might promote an inflammatory environment. In contrast, AuPEI induced a rather immunosuppressed TME. Such dichotomy might arise from the presence of gold in the nanosystem which might counteract any effect from PEI. This is in disagreement with other PEI-coated nanoparticles, such as iron oxide nanoparticles, where the collective effects are conveyed in the pro-inflammatory environment in vitro and in vivo [23], indicating that not only the coating matters but also the core when it comes to the immune system modulation.

## 5. Conclusions

We collectively profiled the immunological microenvironment of the murine mammary MET-1 tumor model upon intratumor injection of PEI-coated AuNPs. By analyzing both myeloid and lymphoid populations, we observed a clear bias toward the anti-inflammatory microenvironment supported by an increase in M2/M1 balance, an increase in PD-1^+^ T cells, and a reduction in NK cells. This study highlights the necessity of profiling all new nanoparticles intended for cancer therapy for the global immunological effect within the tumor niche to understand their intrinsic immunomodulatory behavior better.

## Figures and Tables

**Table 1:** List of antibodies used for cytometry analysis.

Antigen	Fluorophore	Clone	Company	Code
CD11b	BV421	M1/70	BD Biosystems	562605
CD11c	BV605	N418	Biolegend/Ozyme	BLE117334
MHC-II	FITC		Cedarlane	CCL8705F
CD45	PECY7	30-F11	BD Biosystems	552848
Ly-6C	APC-Cy7	HK1.4	Biolegend/Ozyme	128025
CD4	BV 711	GK1.5	BD Biosystems	563050
CD8a	PerCP-e710	53–6.7	eBioscience	46-0081-82
TCRβ	BV605	H57-597	BD Biosystems	562840
Nkp46	eFluor660	29A1.4	eBioscience	50-3351-82
PD-1	BV421	29F.1A12	Biolegend/Ozyme	135217

## Data Availability

Not applicable.
